# Editorial: Ecology, environment, and human microbiome interaction with infection

**DOI:** 10.3389/fpubh.2023.1217927

**Published:** 2023-05-23

**Authors:** Yukiko Koizumi, Fumito Maruyama

**Affiliations:** ^1^Applied Research, American Dental Association Science and Research Institute, Chicago, IL, United States; ^2^Microbial Genomics and Ecology, Center for the Planetary Health and Innovation Science, The IDEC Institute, Hiroshima University, Higashihiroshima, Japan; ^3^Project Research Center for Holobiome and Built Environment (CHOBE), Hiroshima University, Higashihiroshima, Japan

**Keywords:** eco epidemiology, human microbiome, biotic-abiotic interaction with infection pathobiont, dysbiosis, One Health

One Health paradigm, conceived in the early years of the 21st century, encapsulates a global initiative endorsing interdisciplinary collaborations and knowledge dissemination across all dimensions of health sciences. This framework accentuates the intricate interdependencies amongst humans, animals, vegetation, and their shared environment. Ground-breaking advancements in DNA sequencing methodologies and computational biology have significantly transformed the domain of microbiome studies. Analyses of previously uncultured microorganisms deliver exhaustive insights into the associations between animals, the environment, and human microbiota, including various disease implications. Within the context of this investigative topic, we are interested in examining the impact of environmental stressors on bacterial populations and the implications these shifts have for human health.

Environmental fluctuations, inclusive of meteorological variations, influence the propagation of infectious diseases. Liao et al.  delineated the correlation between climatic factors and the emergence of scrub typhus. The authors discovered lagging associations between specific meteorological parameters (rainfall, relative humidity, and ambient temperature) and incidents of scrub typhus that exhibited inverse-U trajectories. The research was oriented toward the development of a preliminary warning system for scrub typhus by harnessing the lagging graphs and associations with meteorological data. Pathogens can be directly transmitted via airborne particles or fomites (e.g., influenza) or indirectly through food, water (e.g., cholera), or a vector (e.g., malaria, dengue), and may involve non-human reservoir species (zoonotic pathogens, e.g., hantavirus). Nagy et al. identified rooks as carriers of extended-spectrum beta-lactamase (ESBL)-producing *Escherichia coli* in a university clinic vicinity. The authors characterized the ESBL gene and established a zoonotic connection between humans and rooks. This study underscored rooks as a long-distance vector that conveys antibiotic resistance to the hospital setting. Antibiotic resistance is universally acknowledged as a pressing One Health concern.

Healthcare-associated infections (HAIs) pose a significant challenge, particularly within hospital environments. These are primarily transmitted via medical equipment, facilities, and healthcare professionals and are often attributable to antibiotic-resistant bacteria. Vancomycin-resistant *Enterococci* and *Clostridium difficile* are the most prominent examples; these tend to originate from environmental sources and subsequently infect the human body. Traditionally, it was presumed that *C. difficile* was primarily hospital-acquired; however, whole-genome sequencing results suggest that the majority of hospital *C. difficile* infection cases originate from external sources/reservoirs that play a vital role in transmission. Tozzo et al. elucidated the localization, transmission, and prevention of ESKAPE species (*Enterococcus faecium, Staphylococcus aureus, Klebsiella pneumoniae, Acinetobacter baumannii, Pseudomonas aeruginosa*, and *Enterobacter species*) and *C. difficile*, the most common pathogens causing HAIs. Tozzo et al.'s review highlighted the fact that microorganisms survive on surfaces for extended periods and that specific cleaning procedures can inadvertently increase the prevalence of pathogenic strains over benign ones. Consequently, treatments that augment populations of beneficial microorganisms, as opposed to those that incompletely clean surfaces, could offer a superior solution to the problem of HAIs.

Patients in ICUs are often prescribed antibiotics, which can annihilate commensal microbiota and therefore escalate the risk of HAIs. Consequently, it is crucial to understand the alterations in commensal microbiota induced by antibiotics and medication profiles in patients. Gage et al. reported that diisopropylfluorophosphate induces a significant reduction in alpha diversity after 48 h, but not 7 days or 5 weeks, in gut microbiota. This study illuminated the relationship between medication, commensal microbiota alterations, and the timing of these occurrences. Scheuring et al. presented a robust system utilizing the Salmoid model to comprehend adaptively significant host–microbe and microbe–microbe interactions. The authors posited that the restoration of healthy microbiota is crucial to averting pathogenic infections. To consolidate our knowledge of healthy microbiota, the identification of pathobionts is integral. Sun et al. discussed the role of human microbiota in the initiation and progression of lung cancer, which is apparent in its induction of inflammatory responses and participation in immune regulation. Furthermore, Yamazaki et al. investigated how the oral pathobionts *Prevotella intermedia* and *Porphyromonas gingivalis*, but not the oral symbionts *Actinomyces. naeslundii* and *Veillonella. rogosae*, exacerbated High-fat diet induced NAFLD, with *P. gingivalis* demonstrating higher pathogenicity. These authors identified potential pathobionts and established their connection with systemic diseases.

To reestablish healthy microbiota, fecal microbiota transplantation has emerged as a more accessible treatment option for those suffering from HAIs and gut dysbiosis, such as inflammatory bowel disease and ulcerative colitis. The dominance of *Candida albicans* in immunocompromised hosts is widely recognized. Munoz et al. developed a probiotic approach utilizing a *Lactobacillus johnsonii* strain (MT4) from the oral cavity of mice and characterized its effect on *C. albicans* growth in planktonic and biofilm states. The authors identified the key genetic and phenotypic traits associated with growth inhibition activity against *C. albicans*. Munoz et al. and Scheuring et al. provided examples of how healthy microbiota can combat nosocomial pathogens.

A microbiota comprises structured multi-kingdom microorganisms, including fungi, archaea, viruses, and bacteria. Current technological advancements facilitate our understanding of inter-kingdom relationships in diseases. Tadmor et al. argued that elucidating the ecology of human-associated phages may have a significant impact on human health because of the potential ability of phages to modulate the abundances and phenotypes of commensal bacteria. They discovered that, despite the great interpersonal diversity observed among human viromes, humans harbor distinct phage families characterized by shared conserved hallmark genes known as large terminase subunit genes. Interestingly, certain phage families were found to be highly correlated with pathogenic, carriage, and disease-related isolates and may serve as novel biomarkers for disease.

In this research area, authors have delineated how environmental shifts precipitate the emergence of infectious diseases. They have further underscored the potential of animals, such as rooks, to carry antibiotic-resistant bacteria to hospitals. Such exogenous resistomes and pathogens contribute to HAIs, which are a global concern. Given that HAIs are not entirely preventable via the cleaning of medical equipment, this topic emphasizes the importance of restoring commensal flora and adopting a probiotic approach to combating nosocomial pathogens, including the identification of pathobionts. The authors have introduced the relationships between animals, the environment, and human microbiota, including various disease correlations.

## Author contributions

Both authors listed have made a substantial, direct, and intellectual contribution to the work and approved it for publication.

